# Classifying Patients with Chronic Pelvic Pain into Levels of Biopsychosocial Dysfunction Using Latent Class Modeling of Patient Reported Outcome Measures

**DOI:** 10.1155/2015/940675

**Published:** 2015-08-18

**Authors:** Bradford W. Fenton, Scott F. Grey, Krystel Tossone, Michele McCarroll, Vivian E. Von Gruenigen

**Affiliations:** ^1^Department of Obstetrics and Gynecology, Summa Health System, Akron, OH 44304, USA; ^2^Division of Cardiovascular Medicine, Department of Internal Medicine, University of Michigan Medical Center, Ann Arbor, MI 48109, USA; ^3^Department of Social and Behavioral Sciences, College of Public Health, Kent State University, Kent, OH 44242, USA

## Abstract

Chronic pelvic pain affects multiple aspects of a patient's physical, social, and emotional functioning. Latent class analysis (LCA) of Patient Reported Outcome Measures Information System (PROMIS) domains has the potential to improve clinical insight into these patients' pain. Based on the 11 PROMIS domains applied to *n*=613 patients referred for evaluation in a chronic pelvic pain specialty center, exploratory factor analysis (EFA) was used to identify unidimensional superdomains. Latent profile analysis (LPA) was performed to identify the number of homogeneous classes present and to further define the pain classification system. The EFA combined the 11 PROMIS domains into four unidimensional superdomains of biopsychosocial dysfunction: Pain, Negative Affect, Fatigue, and Social Function. Based on multiple fit criteria, a latent class model revealed four distinct classes of CPP: No dysfunction (3.2%); Low Dysfunction (17.8%); Moderate Dysfunction (53.2%); and High Dysfunction (25.8%). This study is the first description of a novel approach to the complex disease process such as chronic pelvic pain and was validated by demographic, medical, and psychosocial variables. In addition to an essentially normal class, three classes of increasing biopsychosocial dysfunction were identified. The LCA approach has the potential for application to other complex multifactorial disease processes.

## 1. Introduction

Chronic pelvic pain (CPP) is a syndrome that can encompass one or more underlying pathophysiological processes including the pelvic viscera, muscles, and peripheral or central nervous system [1]. These patients often have other comorbidities such as anxiety, depression, dyspareunia, and sleep disturbances [2]. Overall, the CPP population demonstrates significant impairment in quality of life (QOL) and the ability to function on a daily basis [3]. In an effort to better quantify QOL, the National Institutes of Health (NIH) developed the Patient Reported Outcome Measures Information System (PROMIS), a validated set of multidimensional psychometric assessments (http://www.nihpromis.org/) [4]. The core PROMIS domains provide a method for comprehensively measuring a patient's biopsychosocial function (or dysfunction) in a manner that is broadly applicable to any disease state. The PROMIS system is designed to be independent of the actual nature or number of underlying pathophysiological processes. When the PROMIS metric was used to study women with CPP in a pelvic pain referral center, the PROMIS QOL scores worsened with symptoms of CPP [5].

Given the power of the PROMIS and the 11 domain scores, quantifying CPP into stages of pain appears to be a logical next step in understanding CPP. The benefits of a pain classification system are well known in oncology: planning treatment, estimating prognosis, identifying clinical trial suitability, and providing a nomenclature for comparisons between patients of similar severity [6]. A clinical pain classification system presents a wide range of advantages for practitioners and patients because it defines the extent of a disorder in a manner that is transportable between centers and can be used to measure progression, resolution, and relapse [7]. In addition, a CPP classification system may have clinical applications such as identifying high dysfunction patients appropriate for more intensive treatment, selecting low dysfunction patients appropriate for less invasive approaches, or comparing outcomes using an appropriately weighted stratification scale. The ACTTION-American Pain Society Pain Taxonomy acknowledges current limitations in the understanding of chronic pain and explicitly recognizes the complexity of the “neurobiological, psychosocial, and functional consequences” of chronic pain conditions [8].

The objective of this study was to develop a classification system for CPP based on the core PROMIS measures. In his seminal 1972 paper “More Is Different,” Nobel laureate Andersen posits that systems of increasing complexity cannot be evaluated based on standard reductionist approaches. Instead, “fascinating… and basically new” methods are required to gain a firm understanding of the behavior of these complex systems [9]. Using a latent class analysis of the multiple PROMIS domain scores in female CPP patients to develop a classification system using (LCA) takes a “new” approach to the biopsychosocial consequences of these patients' pain experiences.

## 2. Materials and Methods

### 2.1. Measures

A Summa Health System Institutional Review Board approved protocol permitted measurement of the health status across multiple domains. This was administered via the online PROMIS Assessment Center (http://www.assessmentcenter.net/) to 613 patients referred for evaluation in the Chronic Pelvic Pain Center [5]. PROMIS Assessment Center uses item response theory (IRT) and Computer Adaptive Testing (CAT) to measure well-being in multiple domains in a psychometrically valid, yet efficient manner that selects the most informative set of questions based on responses to previous questions [10]. Patients generated standardized scores for 11 domains of biopsychosocial health: anger, anxiety, depression, fatigue, pain behavior, pain impact, physical function, ability to participate in social activities, social role function, sleep disturbance, and sleep-related impairment.

The latent class measurement model was based solely on the PROMIS domains. Additional patient demographic, medical, and psychosocial measures were collected to further characterize the latent classes identified. Demographic variables included in the analysis were race/ethnicity, age category (5 quantiles), education level, and insurance status. Medical variables studied included smoking status, gravidity (number of pregnancies), parity (number of live births), months of pain (6 quantiles), number of pain-related surgeries, and number of pain-related physicians seen. We employed an abbreviated form of the Childhood Trauma Questionnaire Short Form (CTQ) composed of six items designed to measure sexual, physical, and emotional abuse and physical and emotional neglect [11]. The Posttraumatic Stress Scale (PSS) was used to assess symptoms of Posttraumatic Stress Disorder (PTSD) [12]. The Pain Catastrophization Scale, composed of 13 questions, assessed the amount of magnification, rumination, and helplessness related to pain [13]. The items for each of psychosocial measure were summed for analysis.

### 2.2. Analysis Plan

The analysis sought to identify homogeneous classes of patients across multiple domains of self-reported biopsychosocial health. Patient classification was done by conducting a latent profile analysis (LPA), a type of latent class analysis in which the class indicators are continuous variables like the standardized PROMIS scores [14]. LPA models function differently compared to LCA models with categorical variables in that the underlying joint distribution of the class indicators is multivariate normal, which results in the class indicators having across-class as well as within-class variability which results in covariances of class indicators that can be relaxed or constrained within and across classes, complicating class enumeration. To simplify the LPA to minimize within-class variability and maximize between-class variability so that the standard conditional independence assumption, where almost all class indicator covariance is explained by class membership, holds, the statistical analysis was done in three phases.

First, to minimize within-class variability, an exploratory factor analysis was conducted to identify and combine PROMIS scores that are strongly correlated, as it is known that many of the 11 PROMIS scores are highly correlated with one another. The EFA (results not shown) produced four unidimensional superdomain scores: pain (pain behavior, pain impact), negative affect (anger, anxiety, and depression), fatigue (fatigue, sleep disturbance, and daytime sleep-related impairment), and social function (physical function, ability to participate in social activities, and social role) [1]. Second, the superdomain scores were used as class indicators in the LPA models, and all within latent class covariances were constrained to zero, which assumes that the four domain scores used to classify patients are independent of (uncorrelated with) one another given latent class membership [15]. Third, the variances and covariances across the latent classes were constrained to be zero in the LPA models, which reduced the number of parameters that must be estimated by the LPA, maximized between-class variability, and made class membership the determinant of class indicator variability [14]. Additional models that tested these constraints were run, but as their results did not differ from the simpler analysis, the results are not shown.

To identify the optimal number of latent classes present, LPA evaluated between one and six potential latent classes based on the four superdomains' average scores. A hypothesis based on pelvic pain scores published previously [16] proposed that there would be at least two distinct classes, one representing higher biopsychosocial dysfunction and one representing lower dysfunction. The optimal number of classes was assessed using multiple statistical fit criteria but focused on the Bayesian Information Criterion (BIC) [17] and entropy, which indicates how well patients can be differentiated between classes [18].

After selecting the optimal number of latent classes, CPP patients were staged into their highest probability class. Post hoc descriptive statistics were calculated to produce a demographic, medical, and psychosocial profile for each class. Both EFA and LPA were conducted with Mplus version 7.18 [19] and post hoc analyses were performed using SAS 9.3 (2014, SAS Institute, Cary, NC).

## 3. Results

Determination of the number of latent classes is shown in Table 1, which presents the fit statistics for one through six latent classes. The statistical fit criterion drops substantially with the addition of each latent class until the fifth class is added, at which point these criteria do not change substantially. While Class 6 has the lowest BIC, Class 4 has a higher entropy value as well as a reasonably low BIC compared with a 1- to 3-class system. Taken together, these results led to the adoption of the four-class model.

**Figure 1:**
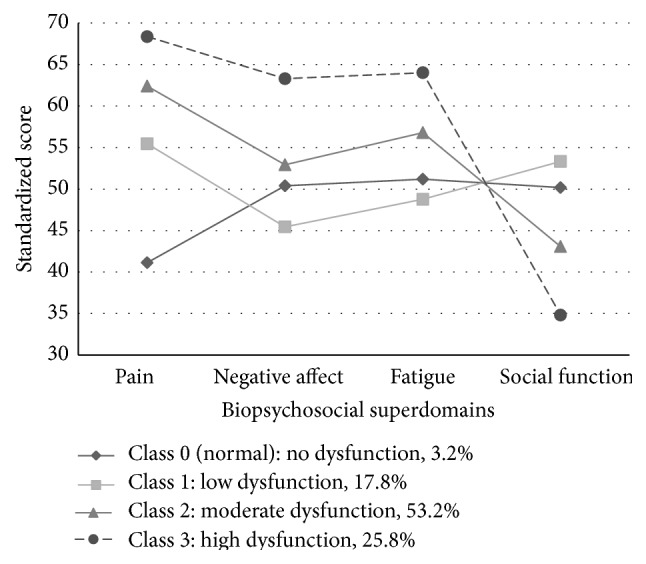
Standardized scores in biopsychosocial superdomains for the four latent classes' model. The PROMIS domains are* t* score adjusted so that the population mean is 50 and 10 points represents one standard deviation. All domains are scored so that higher scores indicate more of what is being measured. For pain, negative affect, and fatigue high scores are worse. For social function low scores are worse. Pain and social function provide the clearest distinction and categorical progression of the latent classes.

Table 2 shows average latent class probabilities for most likely class membership based on the four latent classes' model. This table suggests that between 88 and 92 percent of the sample would be assigned consistently to the same class specification based on class membership likelihood. This indicates a good discrimination of the four-class LCA, as most CPP patients had a high probability of being in a single class with very low probabilities of being in other classes.  Figure 1 graphs the average standardized scores in each of the biopsychosocial superdomains for the 4-class model. The biopsychosocial superdomains, like the original PROMIS measures, are t-scale standardized scores based on a US referent population of 22,000 individuals which produces a mean value of 50 and a standard deviation of 10. As shown in this plot, there is a considerable difference among classes on the four well-being domains, particularly between the first class and the fourth class in this model. This contrast is most obvious in the pain domain, where there is a difference of nearly 30 points between the first class and the fourth class. It is also noticeably different in the social function domain.

Table 3 presents the mean standardized scores and 95% conference intervals for each biopsychosocial superdomain according to class, which coincides with the plot in Figure 1. Given the ordinal distribution of the four superdomains across the classes, four classifications of biopsychosocial dysfunction, labeled 0 through 3, can be proposed: Class 0 (normal): no dysfunction, 3.2% of patients (the “best” class); Class 1: low dysfunction, 17.8% of patients; Class 2: moderate dysfunction, 53.2% of patients; and Class 3: high dysfunction, 25.8% of patients (the “worst” class).

Post hoc demographic characterizations of patients within each of the latent classes are shown in Table 4. Minor differences are seen across the classes, but patients in Class 3 appeared to have received more charity care, have lower levels of education, and are more likely to smoke.  Table 5 contains the medical and psychosocial characteristics across classes. For medical characteristics, there appear to be little differences across the classes, except that patients in Class 0 appear to be more likely to have eight or more pregnancies and to have self-reported months of pain less than three months. For the psychosocial characteristics of the patients, substantial differences are seen across the classes. Class 2 remains close to the mean of all patients for all psychosocial measures (unsurprising as they make up the majority of the sample), while Classes 3 and 4 have substantively lower scores. Class 1 shows substantially higher scores than all the other classes for all three psychosocial measures.

Note that statistical comparisons are not made for post hoc demographic, medical, or psychosocial characteristics across classes. Class membership of patients is assigned based on highest probability, but as standard statistical procedures such as ANOVA assume class membership is known with certainty, such tests will have high type I error rates and therefore are not appropriate.

## 4. Discussion

This is the first application of latent class analysis of patient reported PROMIS measures to evaluate patients with CPP. Using 11 domains from the PROMIS CAT methodology, EFA produced four unidimensional superdomains of biopsychosocial function (pain, negative affect, fatigue, and social function). Two of these (pain and social function) provided an excellent separation of the four classes identified. These findings indicate that patients using the PROMIS approach segregated into four biopsychosocial function groups: Class 3: high dysfunction; Class 2: moderate dysfunction; Class 1: low dysfunction; and Class 0: no dysfunction. The majority of patients (96.2%) had some significant level of pain. Very few (3.8%) reported no pain, and these Class 0 patients are likely similar to healthy, normal women even though they were referred for pelvic pain evaluation [20].

Using the PROMIS for evaluation not only follows current trends in medicine, but until a widely available, noninvasive, and cost effective neuroimaging protocol for pain matrix pathology identification is available, such a system provides a patient-centered approach to comprehensively evaluate a subjective process using an objective system [21]. One of the main advantages of using this approach is that it can account for processes or diagnoses for which there are no currently available evaluation methods. Moreover, these findings may have broad application to both the clinical and research evaluation of patients with CPP. These results, when validated in the larger CPP population, can be used by clinicians to prompt referral for specialized treatment of Class 3 high dysfunction patients and serve as a platform for the standardized communication between clinicians and researchers for describing patients with poorly understood chronic pain pathophysiology.

Due to the complexity of the pain experience and difficulty in predicting outcomes for individual patients, latent class modeling provides a very useful approach to chronic pain evaluation. These would include maladaptive brain-derived neurotrophic factor activity [22], central sensitization [23], or cortically mediated pain [24]. By identifying milder clinical phenomena from more severe cases in areas where the actual disease process is occult, clinicians can select patients for local treatment or referral and can prescribe interventions most likely to be beneficial with the lowest levels of risk.

Clinical phenotyping to predict treatment response is one potential use of LCA. Applying a latent class approach to multidimensional, biopsychosocial QOL and other measures in opiate-dependent patients in Belgium similarly produced a three-class model, with all dimensions clustering together into subjects with low, medium, and high levels of functionality [25]. In cancer patients latent class analysis of patient reported outcome measures was validated across two international populations, compared to an original US referent, and was best fit by a three-class model [26]. Studies such as these indicate the future potential for LCA to prospectively identify patients who may benefit from one particular treatment approach but not another. As suggested by the MAPP research network, many pelvic pain states may share a common underlying central neural pathophysiology, requiring an evaluation of the whole patient rather than just an individual end organ [27].

The characteristics of this patient population and how it segregates into stages (Table 4) provide some insight into the applicability of these findings to other settings. This pelvic pain referral center draws from the area surrounding Akron, OH, USA, and based on the number of previous physicians seen, the number of previous surgeries, and duration of pain, it likely represents a reasonable cross section of patients with a range of severity and chronic pelvic pain. Confirming a range of previous studies on pain and catastrophization [28], traumatic stress [29], and abuse [30], the patients classified to the high biopsychosocial dysfunction class have the highest scores on all of these measures. One hypothesis to account for these findings is that previous experiences and dysfunctional coping mechanisms lead to functional changes in the cortical pain matrix, especially the limbic system [24]. These changes should be evaluable using techniques such as fMRI or distributed source estimation, and patients with higher biopsychosocial dysfunction would be expected to demonstrate more severe coping mechanism dysfunction.

This particular study demonstrates a number of strengths. Although a priori sample size for latent class analysis can be difficult to determine, a general rule of thumb is that, for every statistical parameter estimated (mean, variance, and covariance within and across each class), at least 20 cases must be present. For this study, our sample of 613 CPP patients and the creation of the four superdomains of biopsychosocial function permitted us to evaluate LPA models with up to six classes (including testing the statistical assumptions noted in Materials and Methods). An additional study strength is that it is based on a referral population that has previously been shown to be reflective of chronic pelvic pain patients seen in a general gynecology practice [3]. Also, the PROMIS computerized adaptive testing method was well received by patients: there are no imputed or missing data points.

The use of biopsychosocial function outcome measure based latent class modeling can have a range of limitations. On an allocation level, the mathematical algorithm provides only probability values for any one patient to be in any of the potential classes; thus categorical assignment must possess a nonzero level of ambiguity. The actual analysis is dependent on the population from which it is drawn, and although previously cited studies indicate that there can be substantial stability of latent classes across international regions, this result is not guaranteed without further testing in a broader population of CPP patients. Belonging to a particular class may not provide immediately useful clinical information, and further research is required to understand the significance and utility of class membership. One immediate benefit of using the current PROMIS-based system is that it is free to use through assessment center, and patients classified into the high biopsychosocial dysfunction class may be best managed in a multidisciplinary setting where psychiatry, physical therapy, sleep studies, social work, and pain management can be included in patient care.

These results address the hypothetical question: “Are there measureable classes in patients with CPP?” As a measurement model, the PROMIS is designed only to group patients according to their PROMIS biopsychosocial measure scores. Future work on a predictive model would test the hypothesis that latent class membership can be predicted based on other pertinent neuropsychological tests or physical examination. Although not formally modeled here, the results shown at the end of Table 4 indicate that other historical factors commonly associated with chronic pain, such as catastrophization and abuse, do segregate according to latent class modeled classifications. The most clinically relevant application of the latent class staging system would be an outcome model. This would test the hypothesis that outcomes from different treatments will vary depending on starting class membership. Based on the results shown here, one could posit that patients in the high dysfunction class are less likely to be responsive to treatment than patients in the moderate or low dysfunction classes, when matched for clinical background and treatment type.

The results of this study indicate that patients with chronic pelvic pain can be categorized based on a multidimensional biopsychosocial quality of life scale. This takes into account not just pain and its impact, but also psychological distress, social functioning, and sleep quality. Clinically, these results demonstrate that the PROMIS CAT system is easy to deploy in clinical practice. Patients classified into Class 3, high biopsychosocial dysfunction, are substantially different from patients in the other three classes. This suggests that Class 3 patients are appropriate for more intensive resource utilization such as psychological, physical therapy, or tertiary referral. The research implications are quite broad, including insuring that these classifications are valid at other pain centers, discovering the underling neurophysiology, and developing practical protocols to assist with the clinical management of these complex patients.

## Summary

Latent class analysis of patient reported outcome measures demonstrated four classes of biopsychosocial dysfunction (none, mild, moderate, and severe), validated by pain history and stress.

## Figures and Tables

**Table 1:** Statistical fit criterion for multiple latent class models based on four PROMIS-based superdomains evaluating patients with chronic pelvic pain. The number of classes tested is shown in the first column, and statistical fit parameters for these are shown in the subsequent columns.

Classes	Parameters	Log-likelihood	BIC^1^	aBIC^2^	Entropy
1	8	−6945	13940	13914	N/A
2	13	−6714	13509	13468	0.74
3	18	−6632	13376	13319	0.76
4	23	−6584	13310	13237	0.82
5	28	−6565	13303	13214	0.76
6	33	−6545	13295	13190	0.74

^1^Bayesian Information Criterion. ^2^Sample size-adjusted BIC.

**Table 2:** Average latent class probabilities for most likely latent class membership (row) by latent class (column) according to a four-class model. For each column the probability is shown for the LCA method to assign patients into the different classes. A good model would have a high probability of properly assigning each class (1 s into Class 1, 2 s into Class 2, etc.) and a low probability of misclassification (1 s into Class 2, 3, or 4, etc.).

Class	1	2	3	4
1	0.896	0.000	0.104	0.000
2	0.000	0.879	0.110	0.011
3	0.072	0.047	0.881	0.000
4	0.000	0.078	0.001	0.921

**Table 3:** Model results of a four-class system; means and 95% confidence intervals. The mean score on each PROMIS-based superdomain is shown for each latent class.

Item	Mean	95% confidence interval	
Latent Class 0 (normal)			
No biopsychosocial dysfunction			
Pain	42.40	35.91	48.89
Negative Affect	49.65	34.09	65.21
Fatigue	49.61	35.95	63.27
Social Function	51.74	39.90	63.58

Latent Class 1			
Low biopsychosocial dysfunction			
Pain	55.69	50.69	60.69
Negative Affect	46.76	42.57	50.95
Fatigue	49.57	43.81	55.33
Social Function	52.53	46.36	58.70

Latent Class 2			
Moderate biopsychosocial dysfunction			
Pain	62.63	60.61	64.65
Negative Affect	53.11	49.82	56.40
Fatigue	56.77	54.20	59.34
Social Function	42.82	39.72	45.92

Latent Class 3			
High biopsychosocial dysfunction			
Pain	68.17	66.43	69.91
Negative Affect	63.55	60.67	66.43
Fatigue	64.31	62.23	66.39
Social Function	35.10	33.28	36.92

**Table 4:** Patients' demographic characteristics across class designation. Each individual latent class is described according to demographic characteristics. The characteristics for the entire studied population are shown in the last column. All percentages have been rounded up to the nearest tenth of decimal point and may not add up to 100 percent.

	Class				Total 613
	0: no dysfunction 23 (3.2%)	1: low dysfunction 119 (17.8%)	2: moderate dysfunction 314 (53.2%)	3: high dysfunction 157 (25.8%)	
Race/ethnicity					
Black	7 (30.4%)	17 (14.3%)	56 (17.8%)	30 (19.1%)	110
Hispanic	0 (0.0%)	0 (0.0%)	5 (1.6%)	2 (1.3%)	7
Other	0 (0.0%)	1 (1%)	4 (1.3%)	0 (0.0%)	5
White	16 (69.6%)	101 (84.9%)	249 (79.3%)	125 (79.6%)	491
Age					
14–27 years	8 (34.8%)	49 (41.2%)	94 (30%)	39 (24.8%)	190
28–32 years	4 (17.4%)	16 (13.4%)	89 (28.3%)	29 (18.5%)	138
33–40 years	7 (30.4%)	22 (18.5%)	75 (24%)	51 (32.5%)	155
41–48 years	4 (17.4%)	19 (16%)	41 (13.1%)	26 (16.6%)	90
49–79 Years	2 (1%)	20 (16.8%)	33 (10.5%)	15 (9.6%)	70
Insurance					
None	1 (4.3%)	2 (1.7%)	5 (1.6%)	1 (1%)	9
Private	16 (69.6%)	72 (60.5%)	126 (40.1%)	35 (22.3%)	249
Public	6 (26.1%)	30 (25.2%)	135 (43%)	73 (46.5%)	244
Charity	2 (8.7%)	21 (17.6%)	62 (19.7%)	50 (31.8%)	135
Education					
Less than high school	3 (13%)	3 (2.5%)	35 (11.4%)	25 (15.9%)	66
Graduated HS/GED	5 (21.7%)	53 (44.5%)	139 (44.3%)	82 (52.2%)	279
Associate degree	6 (26.1%)	38 (31.9%)	97 (31%)	39 (24.8%)	180
Bachelor's degree	9 (39.1%)	15 (12.6%)	29 (9.2%)	7 (4.5%)	60
Graduate school	1 (4.3%)	14 (11.8%)	15 (4.8%)	3 (1.9%)	33

**Table 5:** Patients' medical and psychosocial characteristics across class designation. Each individual latent class is described according to medical and psychosocial scores. The characteristics for the entire studied population are shown in the last column. All percentages have been rounded up to the nearest tenth of decimal point and may not add up to 100 percent. CTQ: Childhood Trauma Questionnaire. PTSD: Posttraumatic Stress Disorder Scale.

	Class				Total 613
	0: no dysfunction 23 (3.2%)	1: low dysfunction 119 (17.8%)	2: moderate dysfunction 314 (53.2%)	3: high dysfunction 157 (25.8%)	
Smoker	5 (21.7%)	37 (31.1%)	159 (50.6%)	103 (65.6%)	304
Gravidity					
0 pregnancies	10 (43.4%)	32 (26.9%)	82 (26.1%)	32 (20.4%)	156
1–3 pregnancies	9 (39.1%)	70 (58.8%)	165 (52.5%)	88 (56.1%)	332
4–7 pregnancies	4 (17.4%)	20 (16.8%)	75 (23.9%)	32 (20.4%)	131
8 or more pregnancies	0 (0.0%)	2 (1.7%)	7 (2.2%)	6 (3.8%)	15
Parity					
0 births	11 (47.8%)	36 (30.3%)	107 (34.1%)	49 (31.2%)	203
1–3 births	12 (52.2%)	75 (63%)	194 (61.8%)	96 (61.1%)	377
4 or more births	0 (0.0%)	12 (10.1%)	28 (1%)	11 (7%)	51
Months of pain					
Less than 3 months	1 (4.3%)	3 (2.5%)	9 (2.9%)	9 (5.7%)	22
3 to 6 months	2 (8.7%)	15 (12.6%)	31 (9.9%)	16 (10.2%)	64
6 to 12 months	8 (34.8%)	19 (16%)	64 (20.4%)	26 (16.6%)	117
12 to 36 months	5 (21.7%)	33 (27.7%)	79 (25.2%)	44 (28%)	161
3 to 6 years	4 (17.4%)	28 (23.5%)	65 (20.7%)	27 (17.2%)	124
6 years or more	2 (8.7%)	21 (17.6%)	69 (22%)	35 (22.3%)	127
Number of physicians					
0	3 (13%)	3 (2.5%)	3 (1%)	3 (2%)	12
1	6 (26.1%)	43 (36.1%)	71 (22.6%)	34 (21.7%)	154
2	5 (21.7%)	37 (31.1%)	96 (30.6%)	46 (29.3%)	184
3	2 (8.7%)	14 (12%)	59 (18.8%)	31 (19.7%)	106
4 or more	6 (26.1%)	23 (19.3%)	88 (28%)	44 (28%)	161
Number of surgeries					
0	10 (43.5%)	52 (43.7%)	90 (28.7%)	45 (28.7%)	197
1	7 (30.4%)	28 (23.5%)	79 (25.2%)	41 (26.1%)	155
2	2 (8.7%)	21 (17.6%)	30 (9.6%)	30 (19.1%)	115
3 or more	2 (8.7%)	19 (13.1%)	41 (13.1%)	41 (26.1%)	145
CTQ score^2,5^	2 (3)	2 (3)	4 (5)	6 (6)	4 (5)
Catastrophization score^3,5^	18 (7)	18 (13)	27 (13)	40 (12)	28 (15)
PTSD score^4,5^	6 (14)	7 (7)	14 (11)	25 (13)	15 (12)

^2^Out of a possible 24 points. ^3^Out of a possible 52 points. ^4^Out of a possible 68 points. ^5^Last three rows are the mean (standard deviation).
